# Advancing vaccine development against *Opisthorchis viverrini*: A synergistic integration of omics technologies and advanced computational tools

**DOI:** 10.3389/fphar.2024.1410453

**Published:** 2024-07-15

**Authors:** Alok Kafle, Suvash Chandra Ojha

**Affiliations:** ^1^ Department of Tropical Medicine, Faculty of Medicine, Khon Kaen University, Khon Kaen, Thailand; ^2^ WHO Collaborating Centre for Research and Control of Opisthorchiasis, Khon Kaen University, Khon Kaen, Thailand; ^3^ Department of Infectious Diseases, The Affiliated Hospital of Southwest Medical University, Luzhou, China

**Keywords:** opisthorchiasis, liver fluke, host-parasite, proteomics, immunoproteomics, immunoinformatics, vaccinology

## Abstract

The liver fluke *O. viverrini* (*Opisthorchis viverrini*), a neglected tropical disease (NTD), endemic to the Great Mekong Subregion (GMS), mainly afflicts the northeastern region of Thailand. It is a leading cause of cholangiocarcinoma (CCA) in humans. Presently, the treatment modalities for opisthorchiasis incorporate the use of the antihelminthic drug praziquantel, the rapid occurrence of reinfection, and the looming threat of drug resistance highlight the urgent need for vaccine development. Recent advances in “omics” technologies have proven to be a powerful tool for such studies. Utilizing candidate proteins identified through proteomics and refined *via* immunoproteomics, reverse vaccinology (RV) offers promising prospects for designing vaccines targeting essential antibody responses to eliminate parasite. Machine learning-based computational tools can predict epitopes of candidate protein/antigens exhibiting high binding affinities for B cells, MHC classes I and II, indicating strong potential for triggering both humoral and cell-mediated immune responses. Subsequently, these vaccine designs can undergo population-specific testing and docking/dynamics studies to assess efficacy and synergistic immunogenicity. Hence, refining proteomics data through immunoinformatics and employing computational tools to generate antigen-specific targets for trials offers a targeted and efficient approach to vaccine development that applies to all domains of parasite infections. In this review, we delve into the strategic antigen selection process using omics modalities for the *O. viverrini* parasite and propose an innovative framework for vaccine design. We harness omics technologies to revolutionize vaccine development, promising accelerated discoveries and streamlined preclinical and clinical evaluations.

## Graphical Abstract

**Figure:**
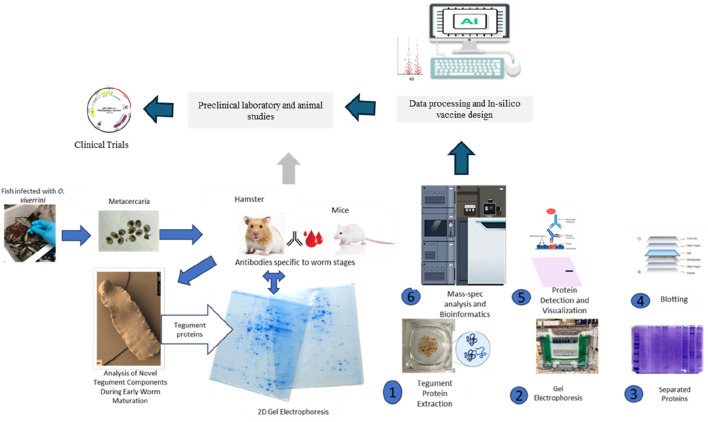
Sincere gratitude to Prof. Jennifer Keiser for generously granting permission for the use of tegument picture in this graphical abstract.

## 1 Introduction

Opisthorchiasis caused by *O. viverrini* (*Opisthorchis viverrini; Ov*) infection is a significant public health issue in many Southeast Asian countries, including Thailand, Lao PDR, Vietnam, and Cambodia, infecting more than 12 million people (Kaewpitoon et al., 2008; Sripa et al., 2021). The liver fluke infection is caused by eating raw or uncooked fish products, which is common in Thailand’s northeastern and northern regions, particularly in rural areas. Infection progression can result in cholangitis, obstructive jaundice, hepatomegaly, cholecystitis, cholelithiasis, and cholangiocarcinoma (CCA), among other hepatic disorders (Sripa et al., 2007). Biliary damage caused by *O. viverrini* examined in the hamster model showed that the parasite infection can solely cause CCA in the long term (Songserm et al., 2009). Hence, *O. viverrini* and *C. sinensis* liver fluke parasites have been classified as “Group 1” biological carcinogens (World Health Organization, 1994).

Upon ingesting raw infected fish, *O. viverrini* metacercariae excyst in the duodenum. The newly excysted juveniles (NEJs) start to migrate towards the ampulla of Vater, relying on chemotaxis and glycogen reserves for the movement (Tielens et al., 1982). Once within the bile duct, they mature into self-fertilizing adult flukes in the bile ducts, thereby acquiring the reproductive capabilities necessary to release eggs (Harinasuta and Harinasuta, 1984; Orido, 1990). The pathogenic mechanisms of *O. viverrini*-induced CCA (*Ov*-CCA) involve parasite-feeding-triggered inflammation, host-helminth interactions at the tegument interface, and immunopathology driven by secreted ES (Excretory Secretory products) products and EVs (Extracellular Vesicles) effector repertoire (Chaiyadet et al., 2019; Glaser, Gaudio et al., 2009; Sripa, 2003; Sripa et al., 2012).

Understanding parasite biology and host interactions better is imperative to identify new drug targets or potential vaccine candidates. The analysis of genomic (Laha et al., 2007; Young et al., 2014), transcriptomic (Jex et al., 2012), and proteomic (Mulvenna et al., 2010) data of *O. viverrini* has provided us with a deeper understanding of *Ov-*tegument and secreted proteins. These proteins may function both protectively and as immunogens in host-specific interactions. Despite substantial research efforts and omics advancement, no vaccine conferring efficacious protection against trematode infections has achieved the level of effectiveness required for commercial deployment (McManus, 2020). Furthermore, the lack of interest from pharmaceutical industries and attention from health agencies and policies is related to the fact that no vaccine against opisthorchiasis is available for clinical use. This can also be attributed partially to the abundance of proteomics data and findings, yet relatively less focus on filtering and analysing these datas for wet lab and translational investigations. Hence, incorporating immunoproteomics is essential in this context, as it facilitates a filter in the rigorous validation of potential markers, thereby bridging the gap between discovery and clinical verification (Pedersen et al., 2005; Ejazi et al., 2018). Moreover, previous investigations exploring antibody responses in the context of parasitic infections have demonstrated the value and feasibility of such immunoproteomics-based studies (Jedlina-Panasiuk, 2002; Pleasance et al., 2011; Zhang et al., 2015; Driguez et al., 2016).

**FIGURE 1:**
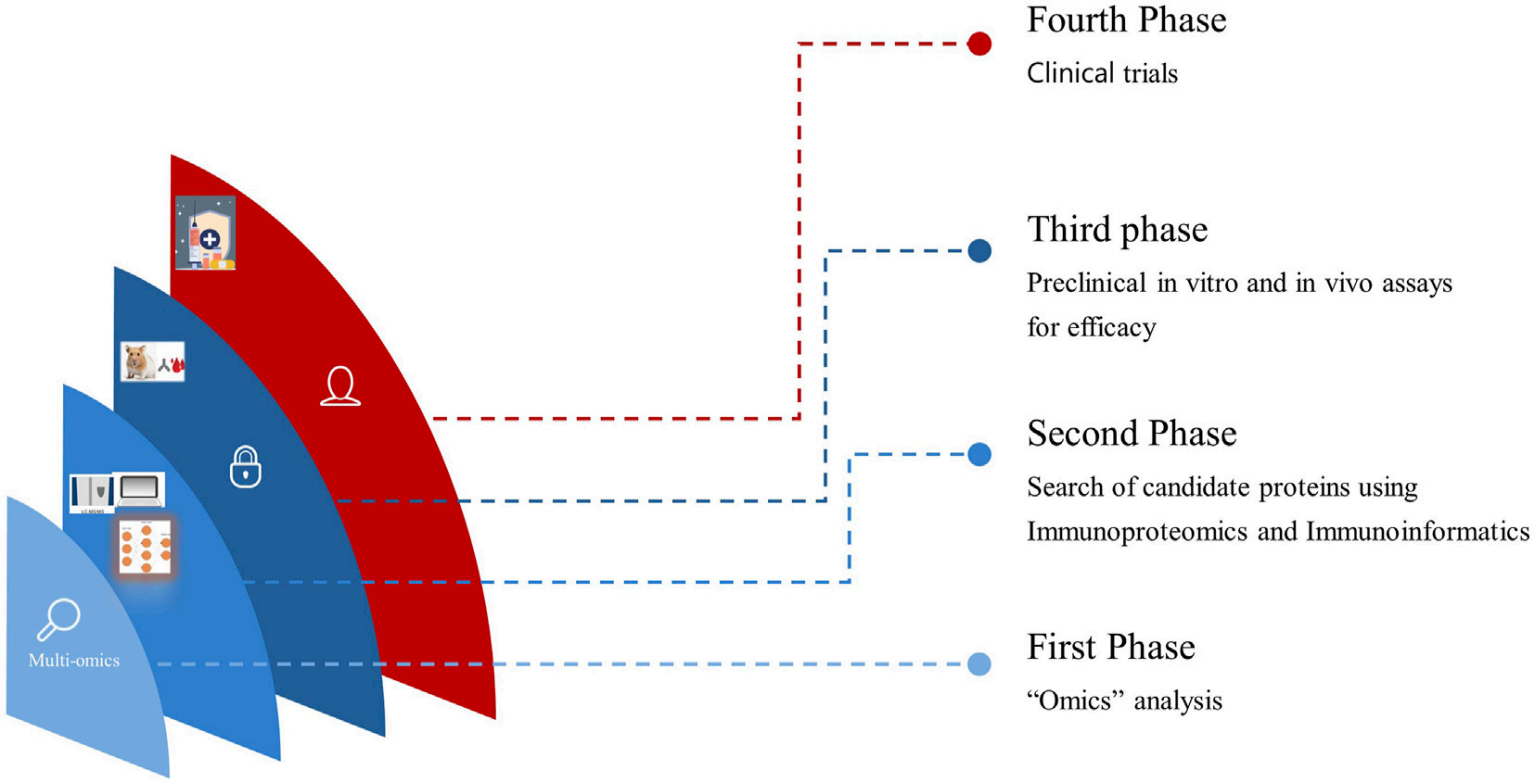
Translational progression by integrating Omics, Immunoproteomics, and *in vitro* and *in vivo* studies seamlessly accelerates vaccine development from discovery to clinical application.

Antigens identified through immunoproteomics analysis spanning from the tegument to ES molecules and EVs of *O. viverrini* can be computationally evaluated. Furthermore, assessing vaccine solubility, antigenicity, immunogenicity, and allergenicity predictions informs the selection of epitopes optimally suited to activating robust host defences (Kapuganti et al., 2022). For the last few years, advanced Artificial intelligence (AI) and Machine Learning (ML) algorithms to predict protein structures and modelling have permitted structural prediction with about 90% accuracy *in silico* (Stevens and He, 2022). Moreover, molecular docking simulations yield insights into projected binding dynamics between prioritised epitopes and immune receptors (Sahoo et al., 2022; Che et al., 2023). Access to these two web-based servers alone holds immense potential to significantly streamline academic research efforts and lead to substantial cost savings. We believe it is now imperative to incorporate immunoinformatics in the prioritisation process before *in vitro* and *in vivo* studies, thereby propelling the progression toward clinical trials more efficiently (Figure 1). Hence, this review highlights the initial phases in vaccine design, which involves the integration of immunoproteomics utilising omics data and computational bioinformatics tools for the systematic studies on helminthic parasites. This integration has the potential to identify numerous protein marker candidates suitable for clinical investigations and to advance the rational design of multi-epitope vaccines against opisthorchiasis.

## 2 Current knowledge of the biology and -omics of *Opisthorchis viverrini* parasite

The multi-omics journey for *O. viverrini* started over 15 years ago by generating and analysing so-called expressed sequence tags (EST) that profiled the first description of gene discovery for the liver fluke *O. viverrini* (Laha et al., 2007). Since 2010, the integration of multi-omics strategies and methodologies, including liquid chromatography-mass spectrometry (LC-MS), next-generation sequencing, and various -omics based technologies, has significantly enhanced our understanding of trematode biology-spanning across the field of genomics, transcriptomics, proteomics, metabolomics, and glycomics (Table 1) (Mulvenna et al., 2010; Jex et al., 2012; Talabnin et al., 2013; Young et al., 2014; Prasopdee et al., 2019; Kokova et al., 2020). Therefore, research efforts focused on the genomes, transcriptomes, and proteomes of *O. viverrini* isolates at a global level hold great potential to significantly advance our understanding of the molecular pathogenesis of *O. viverrini* associated CCA. Such investigations are expected to lay the groundwork for identifying critical targets for designing new interventions.

**TABLE 1:** Overview of omics methods and technologies in *Opisthorchis viverrini* research.

Omics method	Tools/Databases used
Genomics	
Genome Sequencing	Illumina HiSeq2000 sequencing platform
Genome Assembly	SOAPdenovo, Opera, GapCloser, PAGIT iCORN (genome assembly correction)
Repeat and Noncoding Element Identification	RepeatModeler, PILER, LTR_FINDER, RepeatMasker, Repbase library, tRNA-scan-SE, INFERNAL, Rfam database
Protein-Encoding Gene Identification	SNAP, AUGUSTUS, TopHat, Cufflinks, MAKER2, BLASTp, KEGG, OrthoMCL
Functional Annotation	InterProScan, BLASTp (Swiss-Prot, TrEMBL, NCBI non-redundant protein database), GPCR SARfari, Kinase SARfari, KEGG, Transporter Classification Database, Phobius, MultiLoc2
Transcriptomics	
RNA Sequencing	Illumina RNA-seq
Transcriptome Assembly	TopHat, Cufflinks
Differential Gene Expression Analysis	TopHat, Cufflinks
Functional Annotation	InterProScan, BLASTp (Swiss-Prot, TrEMBL, NCBI non-redundant protein database), GPCR SARfari, Kinase SARfari, KEGG, Transporter Classification Database, Phobius, MultiLoc2
Proteomics	
Protein Extraction	SDS-PAGE
Mass Spectrometry	Liquid Chromatography-Tandem Mass Spectrometry (LC-MS/MS)
Protein Identification	Mascot
Immunoreactive Band Excision	LC-MS/MS
Functional Annotation	InterProScan, BLASTp (Swiss-Prot, TrEMBL, NCBI non-redundant protein database), GPCR SARfari, Kinase SARfari, KEGG, Transporter Classification Database, Phobius, MultiLoc2

### 2.1 Genomics and transcriptomics

Before exploring the *O. viverrini* genome and transcriptome, molecular-based studies identified, characterised, and proposed potential drug targets and pathways for exploration. The groundbreaking work of Young et al. (2014) in elucidating the draft genome of *O. viverrini* provided an advanced understanding of the genetic makeup of the liver fluke parasite (Young et al., 2014). This study provided the first global understanding of the biology of *O. viverrini* during its establishment and adaptation to life within the bile duct, helping researchers know how the parasite persists in this unique ecological niche.

Genomic findings provided insights into the developmental stages of *O. viverrini*, revealing that juvenile and immature forms exhibit increased molecular activity and cellular growth. These processes are crucial for their maturation into reproductively competent adults. The expression of distinct genes helps the *O. viverrini* parasite facilitate tissue differentiation, development, and immune evasion, which are functions that become less active in adulthood. The genomic study utilised existing *O. viverrini* transcriptomic data at both the assembly and annotation stages. It continued to map RNA-seq to infer relative expression levels of predicted genes and explore developmental expression changes (Jex et al., 2012). This mapping of transcripts to genes allowed researchers to annotate the predicted functional pathways of these genes based on sequence homology to characterised genes in other species, thereby tentatively linking genes to relevant biological processes such as metabolism, growth, and reproduction, even in the absence of complete ortholog characterisation in *O. viverrini*.

Hence, comparing transcript expression profiles across developmental stages of *O. viverrini* may help identify genes involved in immune evasion and tissue-specific functions, offering potential intervention targets to block the establishment during early parasite infections. Moreover, the transcriptome analysis of adult and juvenile *O. viverrini* worms revealed that immature worms exhibited upregulation in the transcription of peptides associated with energy metabolism. This included oxidative phosphorylation, fatty acid metabolism, and amino acid degradation. In contrast, adult parasites showed increased levels of transcripts involved in nucleotide metabolic processes and processing, oocyte meiosis, and egg-specific antigens. The elevated metabolic activity-related genes observed in the juvenile and immature stages may indicate an anabolic state aimed at acquiring sufficient nutrients from the host to support rapid growth and development. Growth hormones likely play a crucial role in driving tissue proliferation, elongation, and differentiation in juvenile and immature parasites. However, the expression of growth hormones may be downregulated once the adult form is attained. In contrast, mature adults may shift towards a relatively catabolic state focused on survival and reproduction (Jex et al., 2012; Young et al., 2014). Thus, genomic and transcriptomic data availability can help us compare gene expression across developmental stages, reveal the stage-specific biological processes, indicate potential therapeutic vulnerabilities, and offer a more holistic view of the molecular adaptions that enable the parasite’s complex life cycle.

### 2.2 Proteomics

In 2009, Boonmee et al. employed two-dimensional gel electrophoresis to analyse the protein secretion of *O. viverrini* at various maturation stages (Boonmee et al., 2003). These distinctions were identified in the 4-week adult fluke compared to the first-week juveniles, indicating an age-dependent augmentation in protein secretion during the liver fluke maturation. This study provides insights into the protein expression patterns of *O. viverrini* during development, which can significantly contribute to our understanding of the pathogenesis of opisthorchiasis.

In 2010, Mulvenna et al. conducted the first detailed proteomic analysis of *O. viverrini*, using sequential solubilisation of isolated teguments, and a subset of these was localised to the surface membrane of the tegument by labelling *O. viverrini* flukes with biotin and confirming surface localisation with fluorescence microscopy (Mulvenna et al., 2010). This fractionation approach identified five differentially expressed proteins between juvenile and immature stages, of which two were uncharacterised proteins, Experimental autoimmune prostatitis antigen 2 (EAP-2) involved in calcium homeostasis, the cysteine protease legumain, and two granulins that function as growth factors. The study unveiled two granulins, OvGRN-1 and OvGRN-2, as growth factors expressed in both juvenile and adult parasite stages. Alongside identifying various other peptidases and potential markers and targets, their involvement in crucial processes is implied, with certain protein expressions persisting throughout immature and adult life stages (Table 2). This underscores the potential of omics, advanced informatics technologies, and the increasing accessibility of annotated helminth genomes to substantially expedite progress in computational and practical inquiries within NTDs-based studies.

**TABLE 2:** Identifying potential therapeutic targets from -omics data.

Therapeutic targets	Juvenile expression	Adult expression	Potential role
OvGRN-1, Ov-GRN-2, Ov-PRGN Mulvenna et al. (2010), Young et al. (2014)	Yes	Yes	Growth, development and survival. Vaccine candidate, therapeutic target
Cathepsins D Peptidases Mulvenna et al. (2010), Jex et al. (2012)	Yes	Yes	Feeding and movement modulate host immune responses, making it a potential therapeutic target against *O. viverrini*-induced carcinogenesis
GPCRs/Ion Channels Young et al. (2014)	Yes	Limited	Enable chemotaxis-mediated migration of juvenile worms
Antioxidants Young et al. (2014)	Yes	Yes	Protect the parasite by reducing lipid hydroperoxides, a potential therapeutic target.
Phospholipases Young et al. (2014)	Yes	Yes	Play a role in membrane composition and signalling, potential therapeutic target
Lipid-binding Proteins Young et al. (2014)	Yes	Yes	Contains a conserved MD-2-related lipid-binding domain, a potential therapeutic target.
Opisthorchiid-specific galactosylceramidase/galactocerebrosidase and sphingomyelin phosphodiesterases Young et al. (2014)	Yes	Yes	Potential therapeutic target
Legumain Mulvenna et al. (2010), Jex et al. (2012)	Limited	Yes	A cysteine protease is involved in protein degradation, tissue invasion, fibrosis, and cell death
Experimental autoimmune prostatitis antigen 2 Mulvenna et al. (2010)	Limited	Yes	Immune evasion, nutrient acquisition reproduction, and pathogenesis

### 2.3 Glycomics and metabolomics

These omics fields are in their early stages of research, and ample exploration is yet to be undertaken in this domain. *O. viverrini* helminth can express distinctive and highly immunogenic glycans; these glycan antigens share structural features with those present in the intermediate and vertebrate hosts, thus resembling host-like glycans (van Die and Cummings, 2009). The immunogenicity of glycans present on the surface proteins of parasites, especially on the *O. viverrini* tegument, is a critical factor in the parasite’s ability to establish infection and modulate the host immune response (Talabnin et al., 2013). Thus, identifying tegument glycoproteins and their glycan structures in developmental stages can help discover targets that elicit a protective immune response during the early stages of infection (Garcia-Campos et al., 2016).

Metabolomics is another crucial facet of omics research. Studying changes in the parasite’s metabolome during different life stages and in response to the host environment can provide clues about metabolic pathways vital to the parasite’s survival. In the case of *Opisthorchis felinues*, a plasma metabolomics study of the temporal response to opisthorchiasis in an animal model revealed an altered metabolic response in the host, characterised by amino acid depletion and alterations in lipoprotein and cholesterol levels (Kokova et al., 2020). These alterations likely represent a host defence mechanism against the infection, offering perspectives/understanding that could guide therapeutic approaches.

**FIGURE 2:**
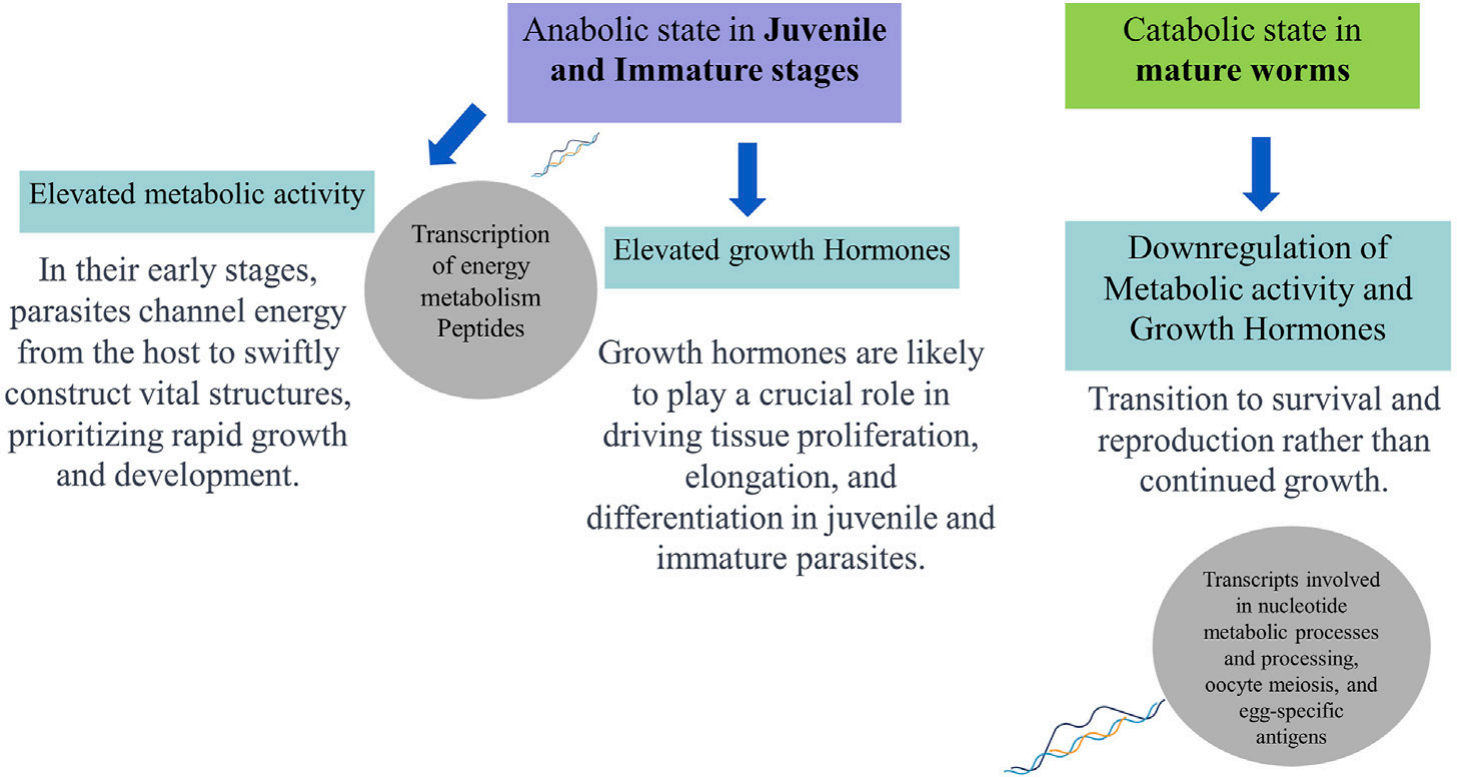
Schematic representation of hypothesised developmental (stage-specific) metabolic profiles and functions of *Opisthorchis viverrini* based on omics analyses.

Therefore, integrating various “omics” techniques, including genomics, transcriptomics, proteomics, glycomics, and metabolomics, can lead to a more profound understanding of the dynamic interaction between *O. viverrini* and its host. In-depth Omics analyses literature discussed above indicate juvenile parasites’ role in upregulating energy metabolism to support anabolic processes during development, synthesising complex molecules from simpler precursors. Conversely, adult parasites transition to lower metabolic activity and growth hormone expression, prioritising survival and reproduction over continued somatic growth (Figure 2). Hence, immunoproteomic approaches, integrating host serology, capture real-time dynamics in the interplay between fluke antigens and the immune response across infection stages, providing a system-level understanding of liver fluke parasite-regulated immune modulation and complementing proteomics in action.

## 3 Exploring host-parasite interactions: Integrating omics discovery with immunoproteomics

As -omics techniques enable the broad discovery of temporally-resolved molecular alterations underlying host-parasite interactions, disease progression, and therapeutic targets during different stages of parasite infection (Hasin et al., 2017; Mitreva, 2022). Considerable progress in parasitology has been made through generating and interrogating omics data (Gardinassi et al., 2018; Liu et al., 2021; Athieniti and Spyrou, 2023). The term “immunoproteomics” is becoming a mature field with potential impact on developing immunoproteomics-based assays and vaccines (Fulton and Walker, 2013). Immunoproteomics explicitly profiles the host immune recognition of protein antigens, aiding the isolation of immunoreactive proteins and leveraging such data to predict and prioritise optimal vaccine candidates efficiently (Dennehy and McClean, 2012).

Immunocapture of antigenic proteins followed by mass spectrometry has proven an effective strategy for learning vaccine targets from trematode parasites (Bennett and Robinson, 2021). The assays have played a crucial role in identifying antigenic proteins from parasitic organisms, including parasitic trematodes (Reamtong, 2013). They leverage an expanding array of technologies to identify and quantify antigenic peptides and proteins that induce immunity (Hayes et al., 2018).

The immunoproteomics techniques include a wide range of gel-based, array-based, mass spectrometry, DNA-based, and *in silico* methodologies to gain unprecedented insights into disease aetiology and progression and facilitate the identification of novel vaccine candidates and biomarker discovery (Table 3). By comprehensively profiling the host immune response at the protein (antigen) level, immunoproteomics enhances our understanding of pathogenic mechanisms and enables accelerated selection of optimal antigens for rational vaccine design against complex infectious diseases. Therefore, integrating immunoproteomics strategies with omics-related discoveries provides a powerful approach for unravelling the functions of upregulated proteins during the infection and a successful vaccine design.

**TABLE 3:** This table compares the principles, advantages, and limitations and provides an immunoproteomics workflow technique to identify antigens from the liver fluke *Opisthorchis viverrini.*

Technique	Principle	Advantages	Limitations	Example procedure with *O. viverrini*
Antigen capture and mass spectrometry	Immobilise antibodies from infected host serum. Capture *O. viverrini* lysate proteins. Elute and digest captured proteins. Identify antigens by MS.	- High sensitivity. - Ability to detect low-abundance antigens	- Biased towards abundant proteins - May miss some antigens	Immobilize IgG from infected hamster serum. Capture proteins from adult *O. viverrini* somatic worm lysate. Elute and trypsin digest. Identify antigens by LC-MS/MS.
SERPA (Serological Proteome Analysis)	Separate *O. viverrini* lysate proteins by 2D-PAGE. Detect antigens by Western blot with infected hamster serum. Identify spots by MS.	- Potential for identifying multiple antigens. - Relatively simple equipment requirements	- Labor-intensive. - Limited resolution. - Biased towards abundant proteins	Separate *O. viverrini* adult worm lysate by 2D-PAGE. Probe blot with infected hamster serum. Identify antigenic spots by MALDI-TOF MS.
High-throughput proteome arrays	Immobilize *O. viverrini* proteins on slides. Probe with infected hamster serum to detect antigenic proteins	- Ability to screen multiple antigens simultaneously. - Lower sample volume required	- Limited antigen diversity	Print microarray with *O. viverrini* recombinant proteins. Probe with serum from infected hamsters. Detect antigenic proteins with a fluorescence scanner
Phage display	Display *O. viverrini* peptides on phage surfaces. Incubate with infected host serum to isolate antigen-binding phages	- Can identify specific peptide antigens. - High diversity of displayed peptides	- Limited to linear epitopes. - False positives from non-specific binding	Display *O. viverrini* peptides on phage surfaces. Incubate with infected host serum. Isolate antigen-binding phages and sequence the displayed peptides
Shotgun immunomics	Fractionate *O. viverrini* lysate. Subject fractions to antibody affinity purification. Analyse captured antigens by mass spectrometry	- Comprehensive antigen discovery. - No prior knowledge of antigens is required	- High cost and complex data analysis. - Some antigens may be missed due to low abundance or binding affinity	Fractionate *O. viverrini* lysate. Perform antibody affinity purification on fractions. Analyse captured antigens by LC-MS/MS.

### 3.1 Tegument of *O. viverrini* as potential vaccine target and biomarker discovery

Immunoproteomics, while crucial for vaccine development, also plays a pivotal role in biomarker discovery and diagnostics by identifying antigens that elicit immune responses (Costa et al., 2023; Germanó et al., 2022; Shi et al., 2018; Zawistowska-Deniziak et al., 2021). The tegument of *O. viverrini*, a multinuclear syncytium, presents an enticing prospect for vaccine development due to its direct contact with the host bile duct and immune system. Vaccination can effectively stimulate the production of both mucosal and systemic antibodies. Moreover, the anatomical arrangement of *O. viverrini* allows for an immediate response of the tegument muscles to external stimuli such as mechanical pressure, ion gradients, nutrient gradients, and damage by deviating immune response that favours parasite survival in the host or avoiding damage through the release of ES products and EVs from the tegument (Chaiyadet et al., 2017; Sripa et al., 2012; Thompson and Geary, 2003).

The tegument analysis of the array of proteins in *O. viverrini* is crucial for unravelling the intricate host-parasite relationship, enhancing opisthorchiasis diagnosis, and advancing the development of marker based studies, therapeutic target based studies to manage *O. viverrini* infection effectively (Liau et al., 2023; Kafle and Suttiprapa, 2024). Studies using serum from *O. viverrini-*infected animals and humans have shown seroreactivity with antigens of the tegument, eggs, reproductive organs, and secretory products (Wilkins et al., 1999; Kafle and Suttiprapa, 2024). These proteins have the potential to be considered vaccine candidates and are more likely to come into contact with the host and may play roles in the parasite’s survival (Young et al., 2014; Molehin et al., 2022). The scanning electron microscopy of the tegument membrane demonstrated stage-specific morphological alterations on the surface of the *O. viverrini* parasite (Apinhasmit et la., 1993). Moreover, several studies have shown differences in tegument proteins based on the gender of worms (Pérez-Sánchez et al., 2008; Zhang et al., 2013; Winkelmann et al., 2022). Thus providing robust evidence for the pivotal role of tegument proteins in parasite reproduction, signal transduction, nutrition, pathogenesis, and host immune modulation (Dalton et al., 2004; Loukas et al., 2007). These interactions are postulated to occur through the parasites’ outer tegument layer and the release of soluble metabolites (Suttiprapa et al., 2018), and this intricate crosstalk fosters tumorigenesis through complex intercellular communication (Elinav et al., 2013).

Moreover, the surface proteins are often considered promising biomarkers due to their heightened specificity for pathogens, particularly in host-parasite interactions. This specificity reduces the likelihood of cross-reactivity with host proteins, making them valuable targets for diagnostic and therapeutic purposes (Retra et al., 2015; Zhang et al., 2015; de Jong and Kocer, 2023).

### 3.2 Early interaction of *O. viverrini* with the host immune system

The survival and pathogenesis of *O. viverrini* depend on the successful evasion of the host immune response by the juvenile and immature fluke stages, which mature into hermaphrodite adults, eventually causing opisthorchiasis. It has been observed that *O. viverrini* metacercaria possesses several antigens similar to those found in adult worms and their metabolic products (Wongratanacheewin et al., 1988).

Comparing protein profiles of *O. viverrini* metacercariae, NEJs to developing immature and mature worms helps reveal stage-specific protein expression. This analysis using LC-MS and bioinformatics may offer insights into the roles of these proteins in the parasite’s development, survival, and reproduction. Several studies have demonstrated the early detection of antibodies in serum as early as 15 days post-infection, with notable inflammatory cell infiltration and the onset of hepatic pathology observed from the third day following infection (Bhamarapravati et al., 1978; Chawengkirttikul and Sirisinha, 1988). This suggests that NEJs and immature *O. viverrini* flukes interact and activate the host immune system during the initial invasion and establishment phases. Furthermore, the suggestion that *O. viverrini* induces immune-suppressive responses early on may facilitate parasite persistence, thereby contributing to developing hepatobiliary diseases associated with chronic *O. viverrini* infection (Sripa et al., 2018). Such investigations identify host-upregulated proteins and signalling pathways detrimental to the parasite, which is crucial for uncovering potential survival strategies in susceptible hosts.

## 4 Integrating immunoproteomics with immunoinformatics for discovering vaccine candidate

**FIGURE 3:**
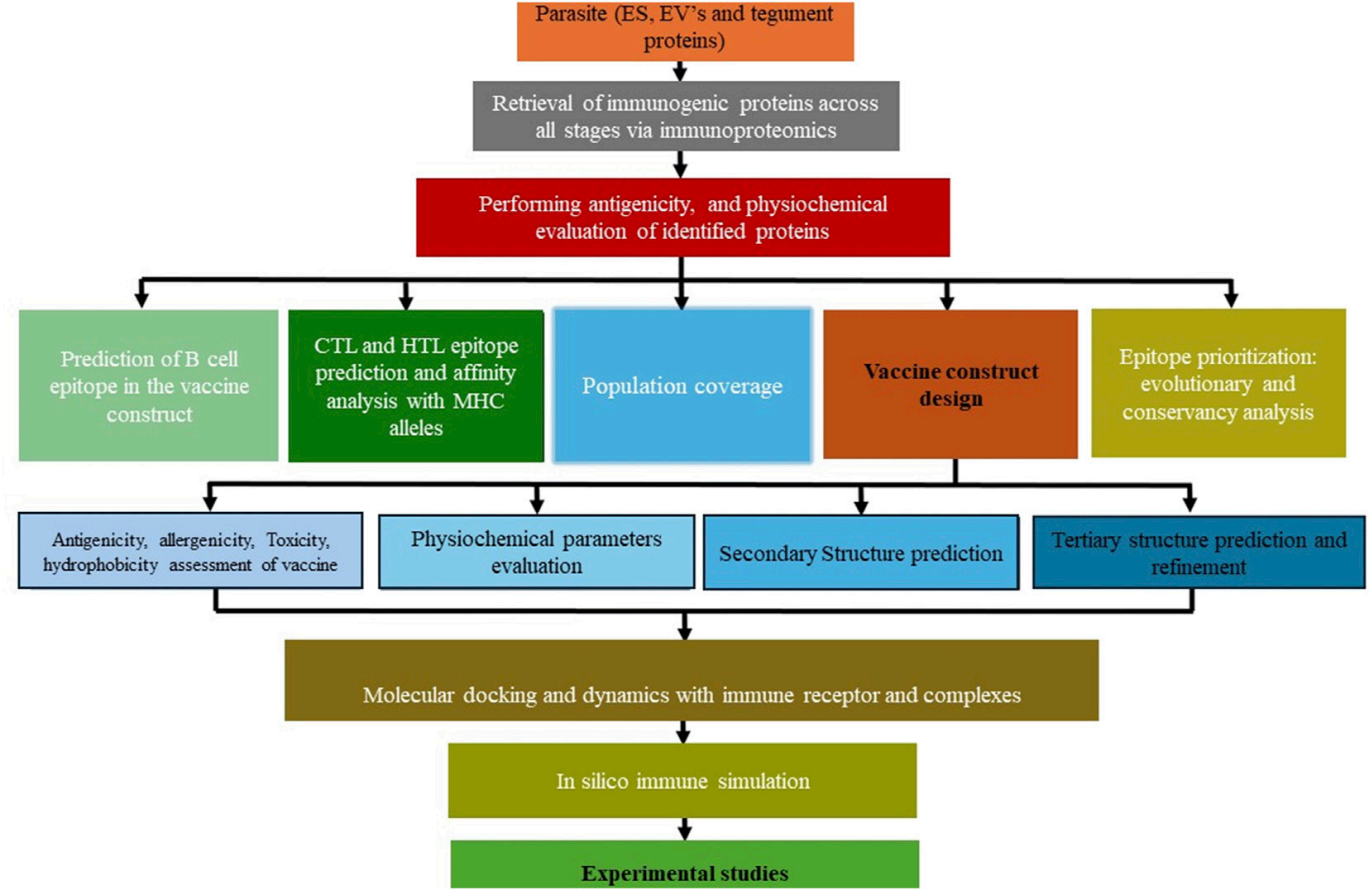
Step-by-step thematic guide for vaccine construction against *Opisthorchis viverrini*.

With the plethora of knowledge in the Omics domain, immunoinformatics, combining immunology and bioinformatics, offers innovative solutions in drug design and identifying vaccine candidates (María et al., 2017; Tomar, 2020). Variations in proteins released in the form of EVs (Trelis et al., 2022) and ES proteins (Wang et al., 2013; Yeshi et al., 2022), and tegument surface proteins may help *O. viverrini* maintain stability, survival, and immune evasion strategies. This can be explored in the systematic vaccine design (Figure 3). Hence, whole proteome analysis in trematode parasitism is beneficial for identifying crucial parasite proteins associated with virulence, exploring essential protein interactions, and uncovering distinctive immuno-modulatory mechanisms contributing to prolonged infection (van der Ree and Mutapi, 2015). Moreover, longitudinal profiling of antibody responses through immunoproteomic tools reveals antigenic proteins expressed in specific parasite developmental stages, from NEJ to immature and adult developmental phases.

**FIGURE 4:**
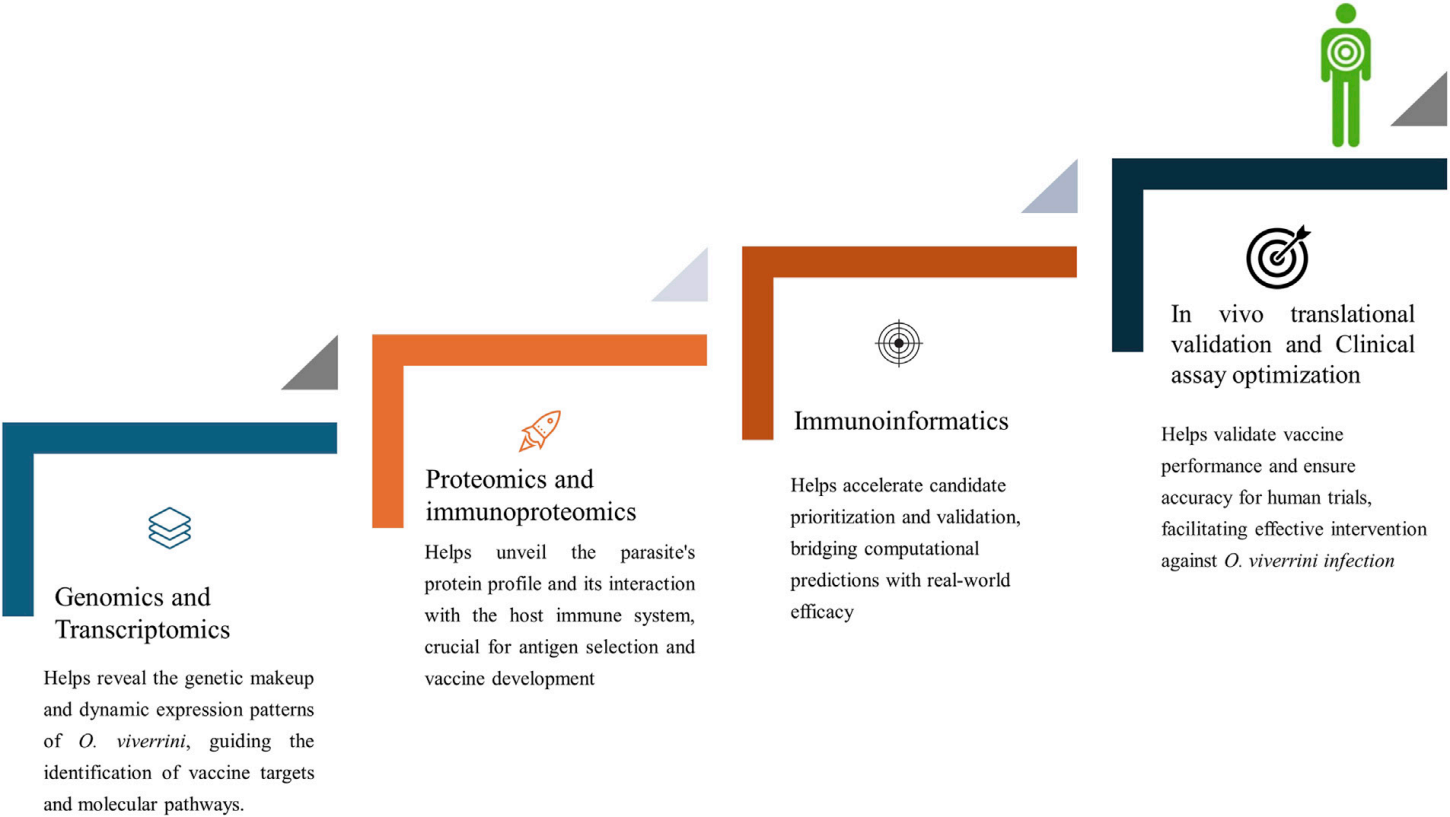
Integrated approaches in vaccine development against *Opisthorchis viverrini*.

The strategy for developing a vaccine against cancer-inducing *O. viverrini* parasites involves using epitopes from antigens to elicit an immune response. However, the primary challenge in creating a cancer vaccine lies in understanding the immunology of infection and identifying inflammation-inducing antigens that can potentially help parasites in vital functions. These challenges can be addressed by blending omics and immunoproteomics results with immunoinformatics (Figure 4).

The *in silico* screening and experimental validation process streamlines identifying and evaluating potential antigens from a liver fluke parasite, offering a faster and more efficient alternative to traditional methods. Candidate antigens can be evaluated *in silico* for population coverage (Bui et al., 2006), antigenicity (Doytchinova and Flower, 2007), physicochemical properties (Wilkins et al., 1999), and ability to induce B- and T-cell epitopes (Bui et al., 2006; Khan et al., 2022). Particularly intriguing are fluke-specific antigens predicted to stimulate both humoral and cellular immune responses in infected hosts, reflecting scientific inclination and necessity. Structural modelling and prediction of immunogenic epitopes using servers like AlphaFold2 (Jumper et al., 2021; Yang, et al., 2023) allows rigorous *in vitro* testing through molecular docking and dynamics to generate hypotheses regarding immune receptors, proteins and designed vaccine interaction (Soleymani et al., 2022). Recombinant protein expression techniques such as cloning predicted multi-epitope subsequences into prokaryotic vectors like pET28b (+) provide a robust means of antigen production and evaluation in immunogenicity studies. (Martinelli, 2022). Various computational and bioinformatics tools and techniques are available in immunoinformatics, particularly in the context of vaccine development (Table 4), which can be utilized for parasitic and infectious diseases.

**TABLE 4:** Comprehensive methodological workflow for vaccine design.

S.No.	Step	Method/Tool used	Purpose/Description	Link
1	Sequence Retrieval and Phylogenetic Analysis	UniProtKB, BLASTx, Clustal W, MEGA	Retrieve the complete amino acid sequence of the identified protein, assess homology, and construct a phylogenetic tree to understand evolutionary relationships	UniProtKB, BLASTx, Clustal W MEGA
2	Antigenicity and Protein Interaction Analysis	Vaxijen 2.0, Topcons, STRING	Determine antigenicity membrane topology and identify protein interactions of paramyosin	Vaxijen 2.0, Topcons, STRING
3	Epitope Prediction	ABCPred, BepiPred-2.0, Discotope-3.0, IEDB	Predict linear B cell epitopes and T cell epitopes, analyse binding affinity to MHC class I and II alleles	ABCPred, BepiPred-2.0, Discotope IEDB
4	Population Coverage	IEDB Population Coverage Tool	Assess population coverage to determine the relevance of the vaccine in regions with high opisthorchiasis rates	IEDB Population Coverage Tool
5	Vaccine Construction	Immunoinformatic Tools, ExPASy Protparam	Design multi-epitope vaccines, incorporate adjuvants, and assess the physiochemical parameters of the constructed vaccine	ExPASy Protparam
6	Protein Structure Prediction and Validation	SOPMA, AlphaFold, Galaxy Refine, ProSA, PROCHECK	Predict secondary and tertiary structures of the vaccine, refine the model, and validate its quality	SOPMA, AlphaFold, Galaxy Refine, ProSA, PROCHECK
7	Conformational B-cell Epitope Prediction	ElliPro	Predict discontinuous B cell epitopes for the vaccine using protein structure approximation	ElliPro
8	Molecular Docking and Simulation	HDOCK, HADDOCK, AutoDock Vina, Amber 22, GROMACS	Investigate protein-protein interactions between the vaccine and TLR receptors	HDOCK HADDOCK AutoDock Vina Amber 22 GROMACS
9	Host Immune Response Simulation	C-ImmSim	Analyse and simulate human immune responses to the vaccine antigen	C-ImmSim
10	In Silico Molecular Cloning	JAVA Codon Adaptation Tool, SnapGene	Reverse-translate and codon-optimize the vaccine sequence, clone it into a vector for expression, and verify the presence of restriction sites	JAVA Codon Adaptation Tool, SnapGene

Note: Numerous web servers and computational tools are updated annually, often with improved versions. When selecting a server, aligning it with your research aim is crucial. However, prioritising tools with the highest prediction accuracy is generally recommended for optimal results.

The synergistic application of experimental vaccine design using omics and computational immunoinformatics represents a promising approach to develop safe, efficacious multi-antigen helminth vaccines rationally. This integrated framework may help overcome decades of challenges by accelerating the selection of optimized antigens that induce robust, broad, and long-lasting immunity against diverse helminth strains. Such vaccines targeting multiple parasite stages and species could significantly reduce the health burden of soil-transmitted helminthiases and other NTDs that predominantly impact low-resource populations worldwide.

## 5 Implication of immunoinformatics in helminth vaccine design

Vaccination has played a pivotal role in mitigating the spread of numerous pathogens and is a cornerstone in disease eradication efforts (Rauch et al., 2018). However, despite its proven efficacy, a notable absence of licensed anti-helminth vaccines for human use remains. Furthermore, helminths, being eukaryotic organisms, present many challenges for vaccination strategies due to their distinct biological characteristics. Hence, immunoinformatics and reverse vaccinology offer transformative avenues in vaccine development. These methodologies leverage computational tools and genomic insights to streamline the identification of potential vaccine candidates from the pool of identified proteins.

Researchers use helminth genomes and proteomes to identify antigenic proteins that initiate immune responses, aiding in the development of customized vaccines. Immunoinformatics methods then prioritize vaccine candidates based on their capacity to elicit protective immune responses, reducing the risk of vaccine failure during advanced clinical trials. Immunoinformatic analysis has been conducted across diverse parasite species. For the focus of our review, we present a curated selection of studies employing the vaccine development pipeline specifically within the domain of helminth parasites, as illustrated in (Table 5). This implies ongoing progress in helminth vaccine research through continual discovery and prioritization of novel candidates.

**TABLE 5:** Immunoinformatics-based identification of helminth vaccine targets.

Author	Helminth species/Disease	Target gene/proteins	Selection techniques/data retrieval	Potential function	Future prospects
Evangelista et al. (2022)	Ascariasis	Piezo protein (two voltage-dependent calcium channels and a protocadherin-like protein)	proteomes predicted from whole-genome sequences of *A. lumbricoides* and *A. suum* for the construction of MEV	Influencing behavior crucial to the parasite lifecycle through calcium-mediated mechanotransduction	Potential for *in vitro*, porcine, and human-based investigations
Akil et al. (2022)	*Fasciola hepatica*	ES antigens (Kunitz-type serine protease inhibitor, cathepsin L1, helminth defense molecule, and glutathione S-transferase)	Protein selection process from previous experimental references proteomes for MEV design	Evading host defenses, facilitating tissue invasion, nutrient acquisition, modulating immune response, and providing protection against oxidative stress	Potential for animal experiments and wet lab studies
Kalita et al. (2020)	*Fasciola gigantica*	FgGST1	Literature review and experimental evidences analysis in the efficacy of GST protein as vaccine targets	Involved in immune defense mechanisms, neutralize the products of lipid peroxidation	Potential for further *in vitro* and *in vivo* assays
Rehman et al. (2021)	Schistosomiasis	Calcium binding and mycosubtilin synthase subunit C	Utilizing Subtractive proteomics pipeline from the reference core proteomes of three Schistosoma species in genome databases for MEV design	Nuclear proteins enter endogenous pathways and serve as class I processing pathway targets	Potential for further laboratory trials
Sanches et al. (2021)	*Schistosoma mansoni*	37 plasma membrane proteins predicted as transmembrane proteins	Using the PSORT II and CCTOP servers for protein selection and MEV design	May participate in functions such as host-parasite interactions, nutrient uptake, immune evasion, or signaling processes	Potential for further *in vitro* and *in vivo* assays
Pandya and Kumar (2023)	*Schistosoma mansoni*	Heat shock protein (HSPs)	Evidence from literature review and experimental evidence analysis in the efficacy of these proteins as vaccine targets	Involved in immune defense mechanisms as they are capable of neutralizing the products of lipid peroxidation	Potential for further *in vitro* and *in vivo* assays
Nourmohammadi et al. (2020)	*Echinococcus granulosus (CE)*	EgA31, EgG1Y162	Literature review, experimental evidence analysis, comparison with known antigens, and consensus building	Vital for the tapeworm’s ability to successfully infect, develop and transmit between its different hosts as part of its life cycle	Potential to assist in future immunization against CE.
Shams et al. (2021)	*Echinococcus granulosus (CE)*	Myophilin	Evidence from previous studies (retrieved as FASTA format *via* the NCBI database)	EgMyophilin expressed in subsegmental parenchyma of PSCs and adult worm suckers helps in mediating host tissue invasion and worm movement by regulating smooth muscle contractions	Potential for *in vivo* validation against CE
Pourseif et al. (2019)	*Echinococcus granulosus*	The sum of *E. granulosus* enolase (EgEnolase) protein sequences	Previous literature and NCBI database retrieval	Key enzyme in glycolysis/gluconeogenesis crucial for parasite invasiveness and virulence	Potential for further *in vitro* and *in vivo* assays
Kaur et al. (2020)	*Taenia solium*	TsM_000971200 and TsM_000948000	Utilised parasite proteomic database (wormbase) to pinpoint vaccine candidates meeting criteria of high antigenicity, non-allergenicity, and membrane localization	Unknown (sequence matched homology with known membrane proteins)	Requires further experimental validation for the safety
Ebrahimi et al. (2020)	*Toxocara canis*	TES-26,TES-30,TES-120	Previous studies and NCBI database retrieval	Obtained from larval culture secretory antigen of T. canis	In vaccines and diagnostic tests
Aarthy et al. (2024)	*Wuchereria bancrofti*	VDM15541 (Kunitz type inhibitor domain-containing protein)	Subtractive proteomics to the entire set of 13,058 proteins linked to W. bancrofti Genome Project PRJEB536	VDM15541, similar to mlt-11 in *Caenorhabditis elegans*, linked to molting, shows strong antigenicity and immunogenicity, being a secretory proteins	Potential candidate requiring experimental validation for the safety and immunogenic behavior
Madanagopal et al. (2023)	*Brugia malayi*	8 highly antigenic proteins (glutathione S-transferase, embryonic fatty acid-binding protein, thioredoxin, abundant larval transcript-2, venom allergen antigen-like protein 1, peroxiredoxin 1, cuticular glycoprotein gp29, and transglutaminase)	Literature findings and retrieved amino acid sequences as FASTA format *via* the NCBI database	Proteins participate in detoxification, fatty acid transport, redox homeostasis, larval development/metabolism, immune modulation, antioxidation, cuticular integrity, and protein bonding	Multi-epitope peptide-based vaccine for LF for animal and human trials
Abraham et al. (2021)	*Onchocerca volvulus*	Ov-103 and Ov-RAL-2	Evidence from previous studies identified and analyzed its protection against the parasite in mouse models	Ov-103 and Ov-RAL-2 antigens, identified in L3, are thought to induce protective immunity *via* an ADCC-dependent mechanism involving crosstalk between IgG antibodies and immune cells	Potential clinical applicability in mice, cows and non-human primates

MEV: multi-epitope vaccine, *Ascaris lumbricoides*: *Ascaris lumbricoides*, *Ascaris suum*: *Ascaris suum*, Fh, fasciola hepatica; GST: Glutathione S-Transferase, EG, Echinococcus granulosus; CE, cystic echinococcosis; PSCs, protoscoleces; TES, toxocarna excretory secretory products; LF, lymphatic filariasis.

## 6 Future direction and conclusion

The lack of attention, prioritization and funding of helminth infections within health organisations and research spheres can be attributed to the absence of opisthorchiasis vaccines currently available for clinical use. Recent strides in transcriptomics, genomics, and proteomics have shed light on the potential engagement of genes and proteins during the early stages of *O. viverrini*, influencing aspects such as survival, motility, immune evasion, and pathogenicity. However, to obtain a clear view of antigen targets, it is crucial to integrate data across various “omics” modalities (Vahabi and Michailidis, 2022), and immunoinformatics represents a sensitive high-throughput platform for profiling immunogenic antigens from the parasite. Insilico analysis with high predictive outcomes can limit the use of animal models, leading to more sustainable research (Pappalardo et al., 2019). In the future, there is hope that *in silico* trials can replace human trials (Ebert et al., 2021). Furthermore, *in silico* analyses in the *O. viverrini* vaccine design process can significantly reduce costs and expedition timelines. Computational screening of antigen candidates can yield substantial savings by minimising experimental wet-lab validation and animal trials, thereby mitigating financial barriers for this under-resourced yet high-impact disease (Saldanha et al., 2023). This approach expedites the entry of numerous vaccine candidates into clinical trials, often ahead of schedule (Shawan et al., 2023). However, the vaccine design against carcinogenic parasites like *O. viverrini* must carefully account for the inflammation associated with its infection and the potential presence of undiagnosed tumours in endemic populations. This consideration is vital due to the intricate interplay between tumours and immune cells, whereby various pathways influence each other, ultimately steering toward distinct outcomes (Gonzalez et al., 2018; Bedognetti et al., 2019). Multistage vaccines have been proposed as an effective strategy in complicated pathogens, as this may show a synergistic effect and would be particularly desirable for biliary tissue-dwelling pathogenic flukes (Maizels, 2021). Furthermore, the choice of proper and safe synthetic immunomodulatory adjuvants should be carefully selected/designed (Zhao et al., 2023) and follow-up *in vivo* studies. Overall, combining immunoproteomics and immunoinformatics offers a compelling strategy to accelerate the rational design and development of vaccines, holding significant potential for preventing and controlling infectious diseases in forthcoming years.

## 7 Implications and limitations

The current period marks an exciting phase for research in this field, with significant advancements seen in recent years, particularly in the structure-based design of new antigens, antibodies, and binders. Traditional vaccine development faces significant challenges, including high costs, hesitancy, stringent safety requirements, and societal demands for perfect efficacy. Additionally, issues like immunosenescence, limited adjuvants, and concurrent health problems in the developing world complicate efforts. Therefore, the global availability of *in silico* technologies in vaccine design and access to tools utilising supercomputers *via* internet-enabled interfaces maximises their utilization across diverse research communities internationally. In NTDs based research, merging AI and ML with biological, medical, and biochemistry-based approaches holds immense promise for driving breakthroughs. This convergence is poised to accelerate the discovery of novel biologics, offering new avenues for combating such neglected diseases effectively (Rodrigues and Ersching, 2014; Winkler, 2021). This initiative aims to end the neglect and contribute to achieving the Sustainable Development Goals (World Health Organization, 2020).

While converging experimental and computational vaccine design holds promise for rational helminth vaccine development, certain limitations must also be acknowledged. These *in silico* based tools require high-quality input data to train algorithms and predict outcomes accurately. Further experimental validation is advised to refine helminth vaccine formulations before proceeding to clinical testing. Moreover, ensuring vaccine effectiveness and safety profiles suitable for use in endemic areas where NTDs are prevalent and infrastructure is limited poses difficulties. In summary, while integrating immunoproteomics and immunoinformatics holds promise for optimising vaccine design, transitioning from theoretical constructs to practical, field-ready vaccines for helminth diseases remains a formidable long-term challenge. Overcoming both natural and technical barriers necessitates sustained dedication from all stakeholders involved.
